# Innovative Application of Probiotic Bacteria to Reduce Dental Plaque in Dogs: A Pilot Study

**DOI:** 10.3390/life16091501

**Published:** 2026-09-08

**Authors:** Anna Misztal-Kunecka, Aleksandra Podgórska

**Affiliations:** 1 Veterinary Clinic DentalVet Anna Misztal-Kunecka, 71-795 Szczecin, Poland; 2 Veterinary Clinic TENVET, 31-586 Kraków, Poland

**Keywords:** dog, dental plaque, probiotics, periodontal disease prevention

## Abstract

Periodontal disease is highly prevalent in dogs and is closely associated with the composition and functional characteristics of the oral microbiome. However, clinical evidence regarding the efficacy of targeted canine probiotics in preventing post-procedural plaque accumulation remains critically limited, representing a significant knowledge gap in veterinary dentistry. In this randomized pilot study, the aim was to evaluate the efficacy of probiotic supplementation in reducing dental calculus accumulation and alleviating clinical signs of oral inflammation in dogs. Statistically significant differences were observed between the study and control groups for all evaluated oral health parameters. The Gingival Bleeding Index (GBI) was considerably lower in the probiotic-treated group than in the control group (*p* = 0.008). Similarly, significant differences were found for the Dental Plaque Index (*p* = 0.020) and the Dental Calculus Index (*p* = 0.018). These results indicate that, after 56 days of probiotic administration, the dogs receiving the probiotic preparation exhibited noticeably better oral health than those in the control group. These observed clinical effects may be consistent with mechanisms proposed in the previous probiotic literature, such as competitive inhibition of biofilm-forming bacteria; however, direct microbiome modulation was not experimentally assessed in this study. The results suggest that probiotic supplementation may represent a promising adjunctive strategy in canine dental prophylaxis by limiting dental calculus accumulation and reducing clinical signs of oral inflammation. These findings provide preliminary clinical evidence of a potential adjunctive benefit, suggesting that post-procedural probiotic administration may support plaque control and help alleviate early signs of oral inflammation in dogs.

## 1. Introduction

The characterization of the canine oral cavity has advanced significantly in recent years, moving away from historical human benchmarks toward culture-independent, high-throughput metagenomic sequencing. These advanced molecular methodologies have successfully mapped the highly complex and distinct nature of the canine oral microbiome under physiological and pathological conditions. It is now well established that periodontal disease—the most prevalent oral affliction in companion animals—is driven by a progressive ecological shift within this microbiome, characterized by a transition from mostly saccharolytic Gram-positive commensals to highly inflammatory, proteolytic Gram-negative anaerobes. Effectively managing this dysbiosis represents a major hurdle in contemporary veterinary medicine as conventional mechanical plaque control protocols suffer from extremely low owner compliance and rapid post-procedural bacterial recolonization. Consequently, there is a high demand for innovative supportive strategies. Microbial therapeutics, including target-specific probiotics and postbiotics, have recently emerged as a promising adjunctive approach capable of actively disrupting pathogenic biofilms and modulating local mucosal immunity without the risks associated with conventional antimicrobial therapies [1,2]. The composition of the oral microbiome can vary considerably between individuals depending on health status, lifestyle, and numerous other factors, such as the use of oral hygiene products (e.g., toothpaste and mouthwash) and inappropriate diets rich in sugars and carbonated beverages. Despite the extensive knowledge of the human oral microbiome, relatively little is known about the composition and function of the oral microbiota of companion animals, which is largely determined by the habits of their owners [3].

Disruption of the oral microbiome is one of the factors that contribute to the development of periodontal disease, which is one of the most frequently diagnosed oral afflictions in dogs [4]. It is estimated that clinical signs of periodontal disease are present in up to 90% of dogs over two years of age, with the prevalence increasing with age and being particularly high in small and toy breeds [5,6]. Periodontal disease can be effectively controlled through regular home oral hygiene performed by the owner. However, the difficulties associated with this procedure, including the time required, poor acceptance by the animal, and anatomical features of toy breeds that make tooth brushing more difficult, result in home oral hygiene being implemented only infrequently [7]. Even regular professional dental cleaning performed in a veterinary practice is insufficient, as dental plaque and calculus begin to accumulate within a few days after the procedure, and gingivitis can develop within a few weeks [8].

An alternative to daily tooth brushing is passive oral hygiene. This includes feed additives, dental chews, and water additives. According to published studies, these alternative methods are effective in controlling periodontal disease in dogs, provided they are used consistently [9]. However, pet owners often regard these products as dietary supplements rather than components of oral hygiene, reducing their effectiveness.

In recent years, modulation of the oral microbiome has emerged as a promising third approach to the prevention and control of periodontal disease. The bacterial biofilm present in the oral cavity plays a key role in the pathogenesis of gingivitis and periodontal disease. Disruption of the microbial balance promotes colonization by potentially pathogenic bacteria, which induce a local inflammatory response in the host. An alternative approach involves the use of probiotics, live microorganisms that confer a health benefit on the host when administered in adequate amounts and with an appropriate composition. Their mechanisms of action include competition with pathogenic bacteria for adhesion sites, production of antimicrobial substances, modulation of the immune response, and stabilization of the local microbiota. In human medicine, selected probiotic strains have been shown to reduce dental plaque accumulation, halitosis, and periodontal inflammation [10]. However, clinical evaluations verifying the practical effectiveness of these biotic preparations as immediate post-procedural home-care tools remain limited. Specifically, whether orally administered strains with transient oral exposure can limit rapid post-scaling plaque re-accumulation and control early gingival inflammation in client-owned dogs represents an unresolved clinical question that warrants objective evaluation.

In the present study, a commercially available probiotic preparation was used as a standardized, quality-controlled source of defined bacterial strains. The product was selected due to the confirmed taxonomic identity of its constituent strains, documented clinical efficacy, and verified bacterial count per sachet, ensuring repeatability and reliable results across the study population. The preparation contained inactivated strains of *Lactiplantibacillus plantarum* AMT4 and AMT14, an inactivated strain of *Bifidobacterium animalis* AMT30, including both bacterial cellular components and their metabolites, and viable *Lactiplantibacillus plantarum* CNCM I-3235 at a concentration of 2 × 10^11^ CFU/kg. The specific combination of inactivated strains (which provide immediate postbiotic immunomodulatory substrates) and viable *L. plantarum* providing viable bacteria with the potential for transient oral exposure or colonization, offers a multi-targeted approach that theoretically optimizes local biofilm control.

The probiotic was supplied as a non-encapsulated powder; it was administered twice daily in the dogs’ drinking water or the dogs’ regular diet. This method of administration was intentionally selected as it allows for rapid distribution of bacterial cells and their metabolites throughout the oral cavity during food or water intake, prior to swallowing. Thus, the probiotic preparation exerts a direct local effect on the oral tissues and tooth surfaces before interacting with the distal segments of the gastrointestinal tract. Modification of the canine microbiome begins almost immediately at the local biochemical level [9,10], whereas stable and sustained restructuring of the microbial community develops after a minimum of two weeks of regular administration [11,12,13]. We hypothesized that the daily oral administration of this multi-strain probiotic formulation, allowing for transient oral mucosal exposure during intake, following professional dental scaling and polishing, would significantly reduce dental plaque accumulation, calculus formation, and gingival inflammation compared with a non-supplemented control group.

## 2. Materials and Methods

### 2.1. Animals

A total of 32 client-owned dogs were initially screened for eligibility in this trial. Following the application of the strict inclusion and exclusion criteria, 20 dogs were excluded: 11 individuals with advanced stage III or IV periodontal disease requiring extensive tooth extractions, 6 dogs diagnosed with systemic metabolic diseases (diabetes mellitus or chronic kidney disease), and 3 dogs that had received antibiotic therapy within 30 days prior to the baseline. Consequently, the final group consisted of 12 mixed-breed dogs aged between 3 and 7 years and weighing 5–15 kg (mean age: 5.5 years; mean body weight: 8.6 kg). All dogs were clinically healthy and free of chronic systemic diseases. Considering the minimum required sample size for this pilot study, a group of 12 patients presenting similar oral lesions was considered sufficient to allow reliable conclusions to be drawn. Due to the exploratory and pilot nature of this study, a formal power analysis was not performed. A sample size of 12 dogs (*n* = 6 per group) was determined based on similar initial pilot evaluations in veterinary dental research, focusing on establishing baseline efficacy and effect sample sizes for future large-scale trials. All dogs were privately owned and written informed consent for participation in the study was obtained from their owners. All dental procedures, including periodontal probing, ultrasonic scaling, and polishing, were strictly therapeutic interventions required for the treatment of the dogs’ pre-existing oral conditions. These procedures were conducted during routine veterinary clinical practice. No experimental, painful, or invasive procedures were performed for the sole purpose of this study. The intervention only involved a commercially available oral feed supplement administered in accordance with standard professional use. Consequently, under Polish legislation (Act of 15 January 2015, on the Protection of Animals Used for Scientific or Educational Purposes; Journal of Laws 2015, item 266), formal approval from an Institutional Animal Care and Use Committee (IACUC) was legally exempt. Rigorous ethical standards were maintained, and written informed consent was obtained from all animal owners prior to inclusion. Dogs were qualified for inclusion based on a clinical examination and intraoral radiographic assessment.

Before the study procedures, each animal underwent a complete physical examination, including visual assessment, cardiac and pulmonary auscultation, capillary refill time evaluation, body weight measurement, body condition assessment, and body temperature measurement. Blood samples were collected for routine laboratory testing.

The exclusion criteria concerned health status and radiographic data. Dogs with chronic diseases were excluded because such conditions may influence oral health and prevent reliable comparison with clinically healthy animals. Metabolic diseases may alter the pH of the oral cavity and consequently affect the composition of the oral microbiome, while concurrently administered medications may interact with the probiotic preparation. Therefore, only dogs with an American Society of Anesthesiologists (ASA) physical status classification of ASA II or lower were included. Dogs presenting clinical or radiographic evidence of periodontal disease exceeding stage II, or requiring extraction of teeth included in the evaluation, were also excluded from the study. The evaluated teeth were the maxillary I3, C, P3, P4, and M1, and the mandibular C, P3, P4, and M1, in accordance with the recommendations of the Veterinary Oral Health Council (VOHC). These teeth had to be present and exhibit no evidence of periodontal disease beyond stage PD2 according to the American Veterinary Dental College (AVDC) classification [14]. An initial oral examination was performed in conscious animals to verify the presence of the required teeth and to exclude dogs with obvious signs of advanced periodontal disease. The inclusion criteria were reassessed on Day 0 under general anesthesia, and dogs with periodontal disease exceeding stage PD2 were excluded.

Root canal treatment performed concurrently with the study procedures did not constitute an exclusion criterion. These treated teeth exhibited pulp necrosis, most commonly of traumatic origin in the preoperative and postoperative radiographs. Unlike pulpal gangrene, pulp necrosis does not significantly alter the bacterial flora of the oral cavity; therefore, it was unlikely to influence the study outcomes.

### 2.2. Products and Randomization

The animals were randomly allocated into two groups using a simple computer-generated randomization sequence (generated via online software). Allocation concealment was performed by a veterinary technician not involved in the clinical assessments using sequentially numbered, opaque, sealed envelopes. The first group served as the control group (*n* = 6) and did not receive probiotic supplementation. Dogs assigned to the second group (study; *n* = 6) received the probiotic preparation PetBIOM^®^ (Owlie S.A., Stawiguda, Poland) at a dose of one-half sachet (1.5 g) twice daily. Probiotic administration commenced on the day of the dental procedure and continued throughout the 56-day study period. The dosing regimen (1.5 g twice daily, providing a minimum of 3 × 10^8^ CFU per dose of viable bacteria) was selected to ensure adequate local mucosal coverage based on successful scaling designs in the canine probiotic literature [2,10]. Throughout the study, all dogs were fed the same dry diet (Virbac Adult Small & Toy—Virbac, Carros, France). Oral examinations were performed 4 and 8 weeks after the dental procedure. The observation period lasted exactly 56 days. This standard 8-week timeline was selected to conform strictly with established veterinary dental testing methodologies, such as those recommended by the Veterinary Oral Health Council (VOHC), which utilize 28-day testing intervals (Day 28 and Day 56) to ensure direct mathematical comparability with historical active dental trials. With the owners’ cooperation, all other forms of oral hygiene (including dental chews, tooth brushing, toothpaste, and similar products) were prohibited during the study period. Drinking water, with or without the probiotic preparation, was provided *ad libitum*.

#### Compliance Monitoring

To ensure high-quality data and minimize the risk of protocol deviations related to non-compliance, a monitoring procedure for the pilot research was implemented. Each owner received written instructions describing the administration of the probiotic preparation, including the dosage, frequency of administration, and duration of treatment. In addition, individual verbal instructions regarding administration were provided. Throughout the study, regular telephone contact with the owners was maintained, to monitor adherence to the study protocol and address any questions regarding the intervention. During follow-up visits, owners were required to return the empty sachets of the probiotic preparation, providing an additional means of verifying compliance. Based on the sachet count and owner logs, the overall treatment compliance was 96.4%, with all included dogs receiving at least 90% of the prescribed doses.

### 2.3. Dental Procedure

Periodontal disease (PD) was assessed under general anesthesia on Day 0. The examination included a comprehensive oral evaluation, periodontal probing, completion of a dental chart, full-mouth intraoral radiography, and assessment of the Gingival Bleeding Index (GBI). Dogs diagnosed with periodontal disease exceeding stage II according to the AVDC classification [14] were excluded from the study. The first stage of the procedure consisted of thorough removal of dental calculus and plaque using an ultrasonic scaler followed by polishing with prophylactic cups. The oral cavity was then irrigated with a chlorhexidine solution. When tooth extraction was indicated, it was performed using an open surgical technique. A mucoperiosteal flap was elevated, the buccal alveolar bone was removed while preserving the lingual or palatal cortical plate, and the tooth was extracted. Following extraction, the alveolus was curetted, packed with Surgispon^®^ collagen material, and closed using 4–0 monofilament suture material placed in simple interrupted sutures to ensure tension-free wound closure. These extractions did not require routine antibiotic therapy. Instead, dogs received analgesic and anti-inflammatory treatment with meloxicam (0.1 mg/kg body weight) for four days following surgery. The effectiveness of professional dental cleaning was verified using a plaque-disclosing solution (Plaque IC, IM3 Dental Ltd., Duleek, Ireland) to ensure complete removal of dental plaque and calculus. If residual deposits were detected, ultrasonic scaling and polishing were repeated until complete cleaning had been achieved.

Subsequently, gingival inflammation, dental plaque, and dental calculus were evaluated on the buccal surfaces of six index teeth: the maxillary canine (C), third premolar (P3), fourth premolar (P4), the mandibular canine (C), fourth premolar (P4), and first molar (M1), bilaterally. The Gingival Bleeding Index (GBI) [15] was assessed using a periodontal probe according to the following scale: 0 = no inflammation; 1 = mild inflammation (slight color change, slight edema, and no bleeding on probing); 2 = moderate inflammation (redness, oedema, and delayed bleeding on probing); and 3 = severe inflammation (immediate bleeding on probing). Dental plaque was assessed using a plaque-disclosing solution (Plaque IC, IM3). Plaque coverage was scored as follows: 0 = none; 1 = <25%; 2 = 25–50%; 3 = 50–75%; or 4 = 75–100%. Plaque thickness on the crown was graded as 1 = light, 2 = moderate, or 3 = heavy. The plaque index for each tooth was calculated as the product of plaque coverage and plaque thickness, as previously described [16,17]. Before assessment of dental calculus, dental plaque was removed from the tooth surfaces using a toothbrush. Each index tooth was evaluated individually, and the mean oral scores for the Gingival Bleeding Index, Dental Plaque Index, and Dental Calculus Index were calculated for each dog. Clinical assessments were performed at three time points: Day 0 (D0, immediately after professional dental cleaning), Day 28 (D28), and Day 56 (D56). The primary outcome measure was the reduction in dental plaque accumulation on Day 56, assessed via the Dental Plaque Index. Secondary outcome measures included changes in the Gingival Bleeding Index (GBI) and the Dental Calculus Index evaluated at Day 28 and Day 56.

### 2.4. Statistical Analysis

Statistical analyses were performed using Statistica 13.0 software (StatSoft, Kraków, Poland).

As a first stage, descriptive statistics were calculated for all variables. The results are presented as the number of observations (*n*), mean, median, standard deviation (SD), interquartile range (IQR), minimum value, and maximum value.

The data distribution was formally checked using the Shapiro–Wilk test, which confirmed a non-normal distribution for the majority of the clinical indices. This formal assessment, combined with the small sample size (*n* = 6 per group) and the ordinal nature of the scoring scales, justified the use of non-parametric methods. Owing to the pilot nature of the study, the small sample size (*n* = 6 per group), and the ordinal nature of the clinical scoring systems, non-parametric statistical methods were used for subsequent analyses.

Baseline comparability between the study and control groups (D0) was assessed using the Mann–Whitney U test. The primary efficacy analysis compared the Gingival Bleeding Index (GBI), Dental Plaque Index, and Dental Calculus Index between the probiotic-treated and control groups on Day 56 (D56) using the Mann–Whitney U test.

The magnitude of the treatment effect was expressed as the percentage reduction in each evaluated parameter in the probiotic group relative to the control group.

The results are presented in tabular and graphical form. Differences between groups were visualized using boxplots, whereas temporal changes were illustrated using line graphs. Statistical significance was set at *p* < 0.05.

## 3. Results

### Descriptive Statistics

Table 1 presents the descriptive statistics for the evaluated oral health parameters in the control group (*n* = 6) at the three study time points: immediately after professional dental cleaning (D0), after 28 days of observation (D28), and after 56 days of observation (D56). At baseline (D0), the mean Gingival Bleeding Index (GBI) was 2.17 ± 0.75, the mean Dental Plaque Index was 4.00 ± 1.26, and the mean Dental Calculus Index was 2.50 ± 0.55. Twenty-eight days after the procedure, a marked improvement was observed in all evaluated parameters. The mean GBI decreased to 0.50 ± 0.55, the Dental Plaque Index decreased to 1.67 ± 0.82, and the Dental Calculus Index decreased to 0.67 ± 0.82. On Day 56, partial deterioration of all evaluated parameters was observed compared with Day 28. The mean GBI increased to 1.17 ± 0.41, the mean Dental Plaque Index increased to 2.33 ± 0.52, and the mean Dental Calculus Index increased to 1.33 ± 0.52.

**Figure 1:**
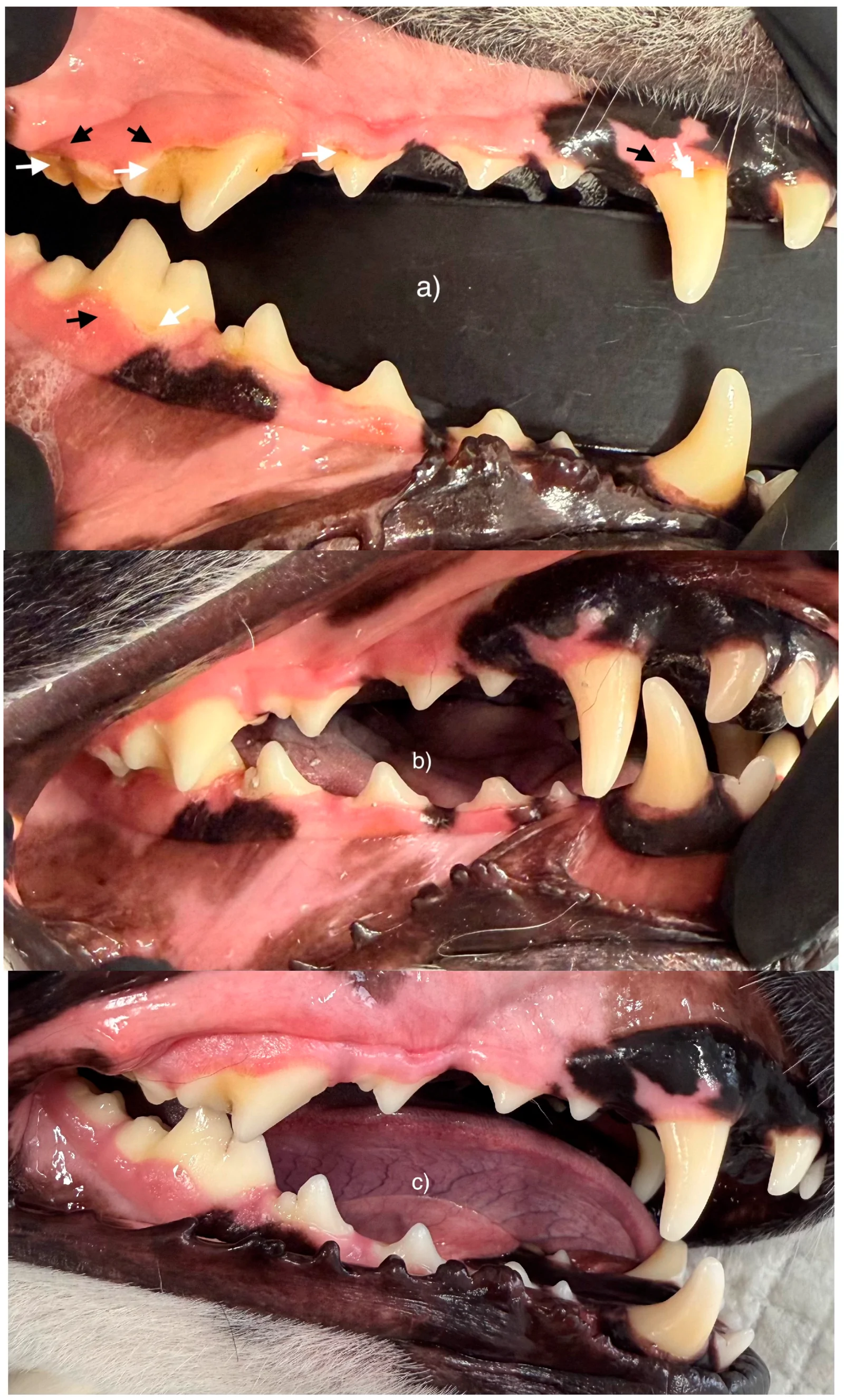
Photos showing the condition of the oral cavity (**a**) D0, (**b**) D28 and (**c**) D56 post-procedure in the group receiving the PetBIOM^®^ probiotic preparation. White arrows indicate dental calculus and black arrows gingivitis.

Table 2 presents the descriptive statistics for the evaluated oral health parameters in the probiotic-treated group (*n* = 6) at the same three timepoints. At baseline (D0), the mean Gingival Bleeding Index (GBI) was 1.83 ± 0.75, the mean Dental Plaque Index was 4.00 ± 1.10, and the mean Dental Calculus Index was 2.50 ± 0.55. After 28 days of probiotic administration, all evaluated parameters showed marked improvement. The mean GBI decreased to 0.17 ± 0.41, the Dental Plaque Index decreased to 0.50 ± 0.55, and the Dental Calculus Index decreased to 0.33 ± 0.52. On Day 56, these values remained low. The mean GBI remained unchanged at 0.17 ± 0.41, while the Dental Plaque Index increased slightly to 1.17 ± 0.75. The Dental Calculus Index remained stable at 0.33 ± 0.52 (Figure 1).

**Figure 2:**
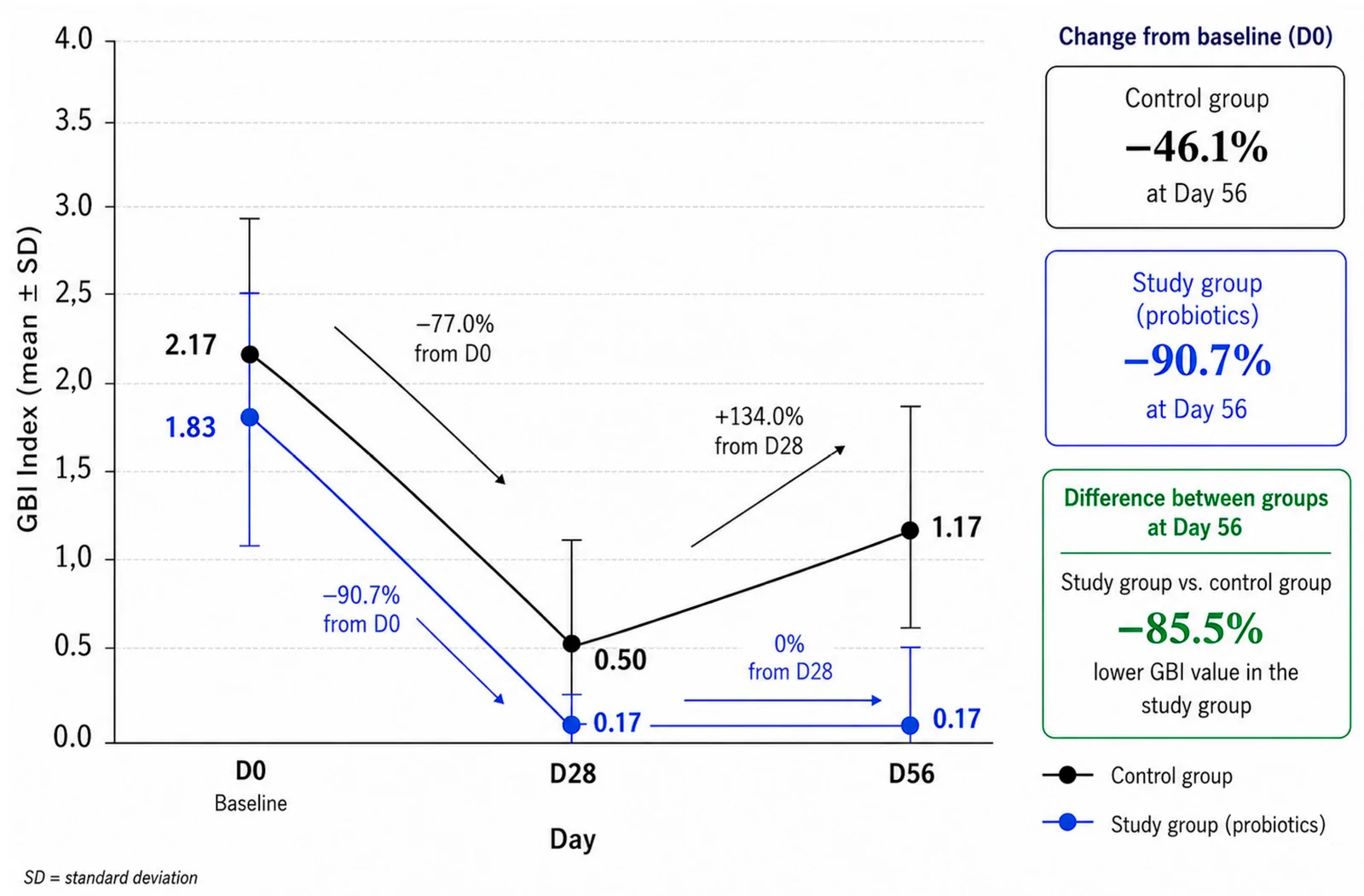
Changes in the mean Gingival Bleeding Index (GBI) values in the control group and the probiotic-treated group at the three study time points (D0, D28, and D56).

Exploratory evaluation of the descriptive statistics suggests a potential trajectory toward sustained beneficial effects of probiotic supplementation on oral health across the study period. The most stable effect was observed for the reduction in gingival bleeding (Figure 2) and dental calculus accumulation, whereas a slight tendency toward renewed plaque accumulation was noted between Days 28 and 56.

Table 3 presents the comparative analysis of the control and probiotic-treated groups at baseline (D0). Group comparability before the initiation of the intervention was assessed using the Mann–Whitney U test. Three oral health parameters were analyzed: the Gingival Bleeding Index (GBI), Dental Plaque Index, and Dental Calculus Index.

**Figure 3:**
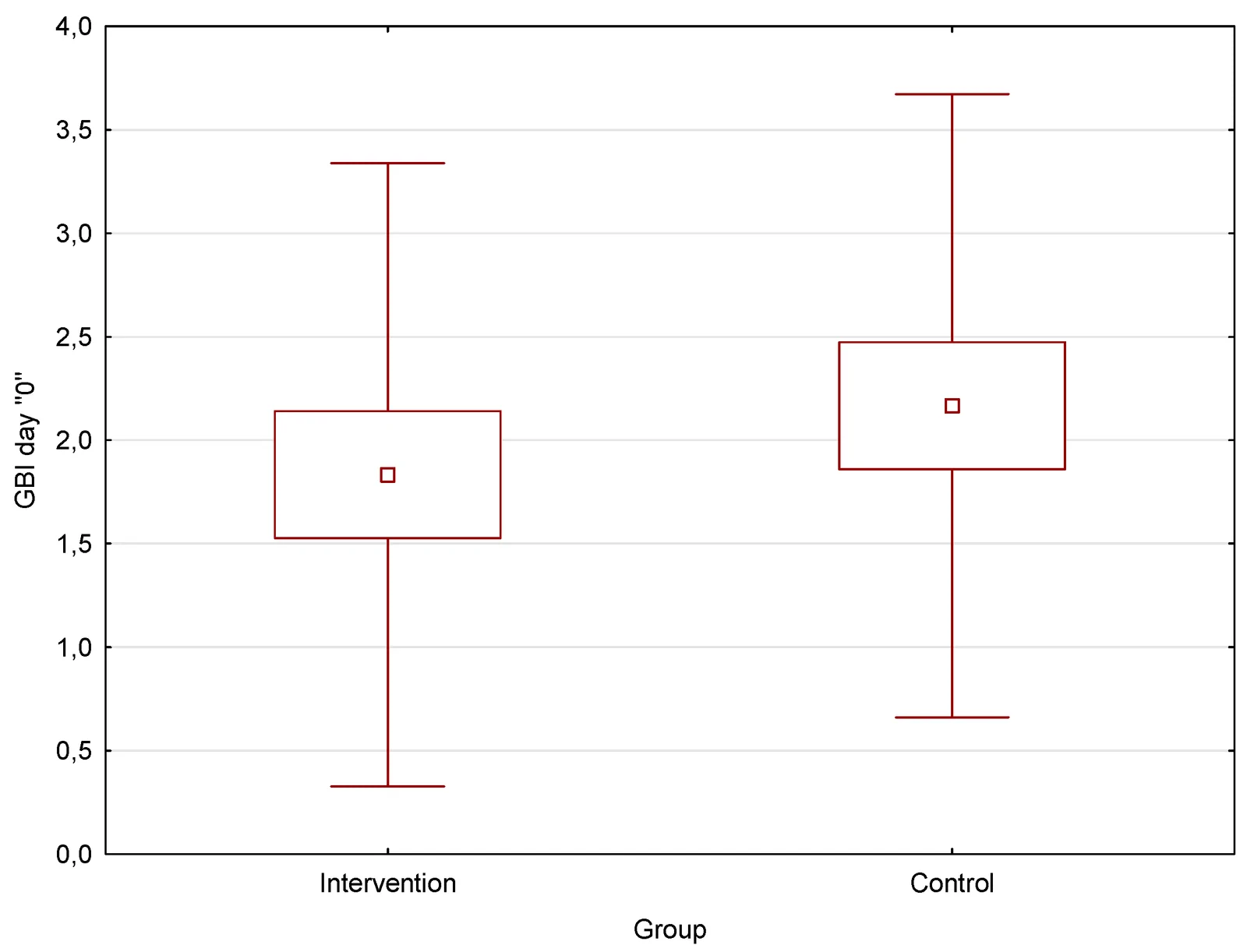
Boxplot showing the distribution of Gingival Bleeding Index (GBI) values in the control and probiotic-treated groups at baseline (D0).

**Figure 4:**
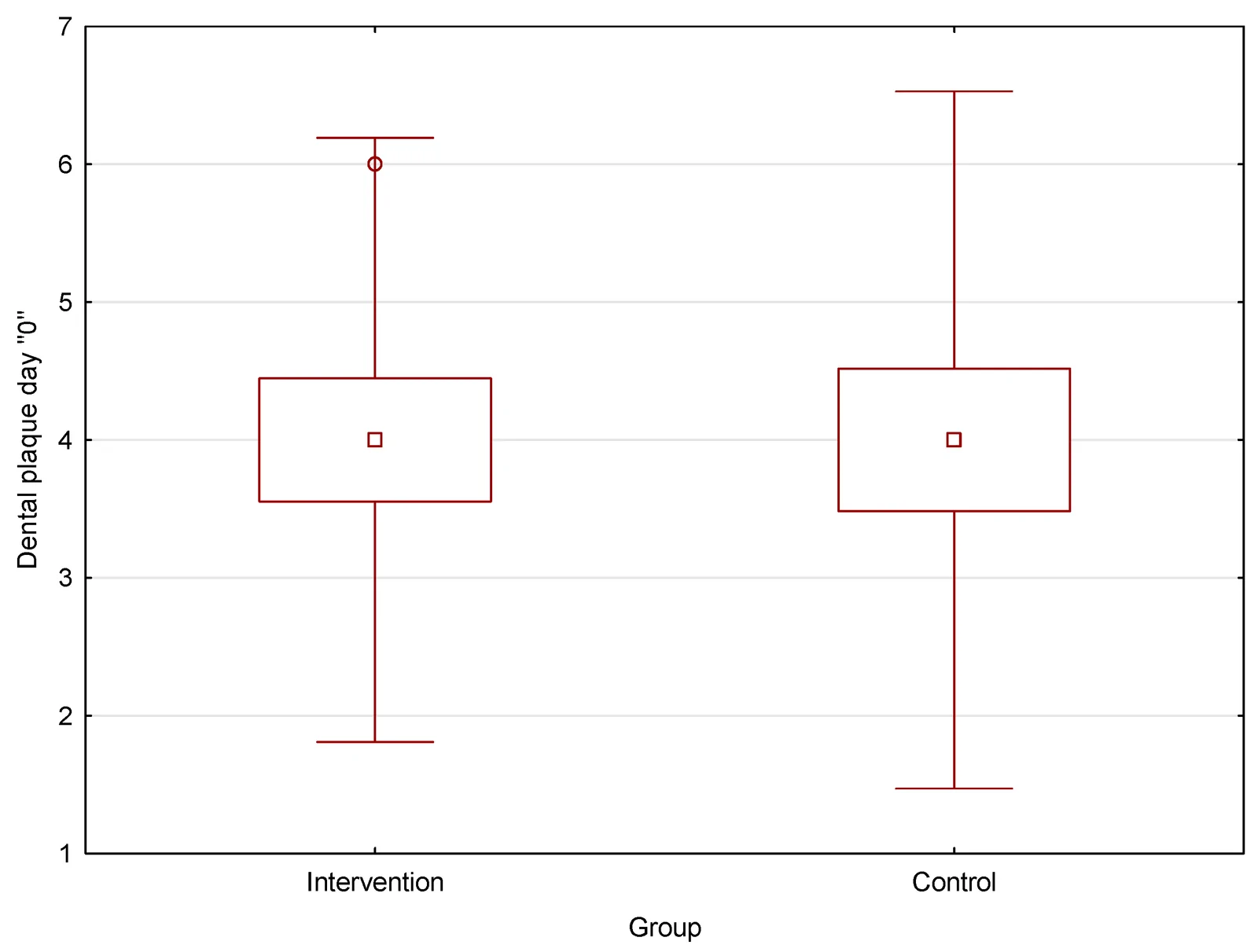
Boxplot showing the distribution of Dental Plaque Index values in the control and probiotic-treated groups at baseline (D0).

**Figure 5:**
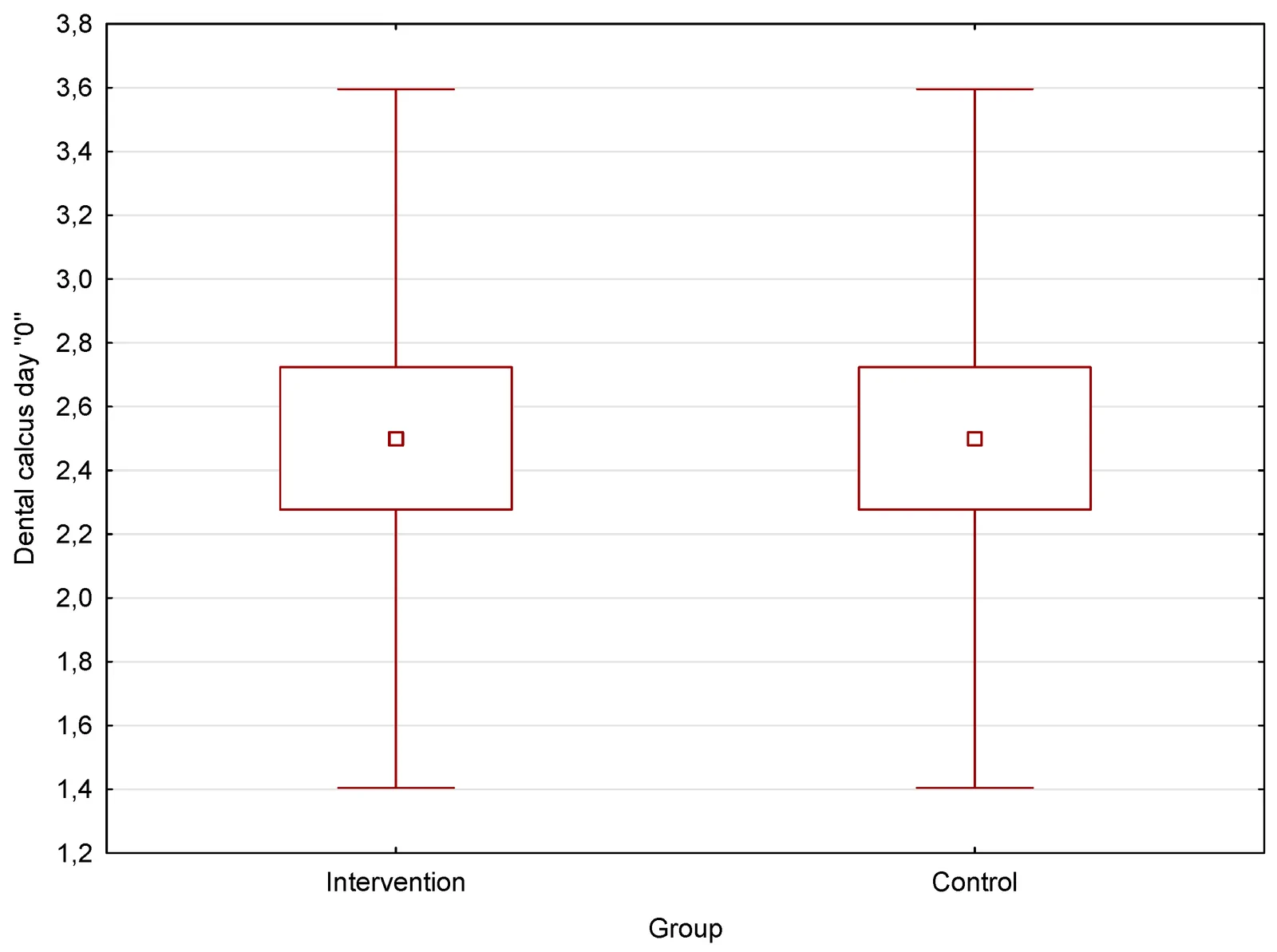
Boxplot showing the distribution of Dental Calculus Index values in the control and probiotic-treated groups at baseline (D0).

No statistically significant differences (*p* > 0.05) were found between the control and probiotic-treated groups for any of the evaluated parameters. The *p*-values were 0.487 for GBI (Figure 3), 0.932 for the Dental Plaque Index (Figure 4), and 0.927 for the Dental Calculus Index (Figure 5).

Table 4 presents the comparison of oral health parameters between the probiotic-treated group and the control group on Day 56 (D56) using the Mann–Whitney U test.

**Figure 6:**
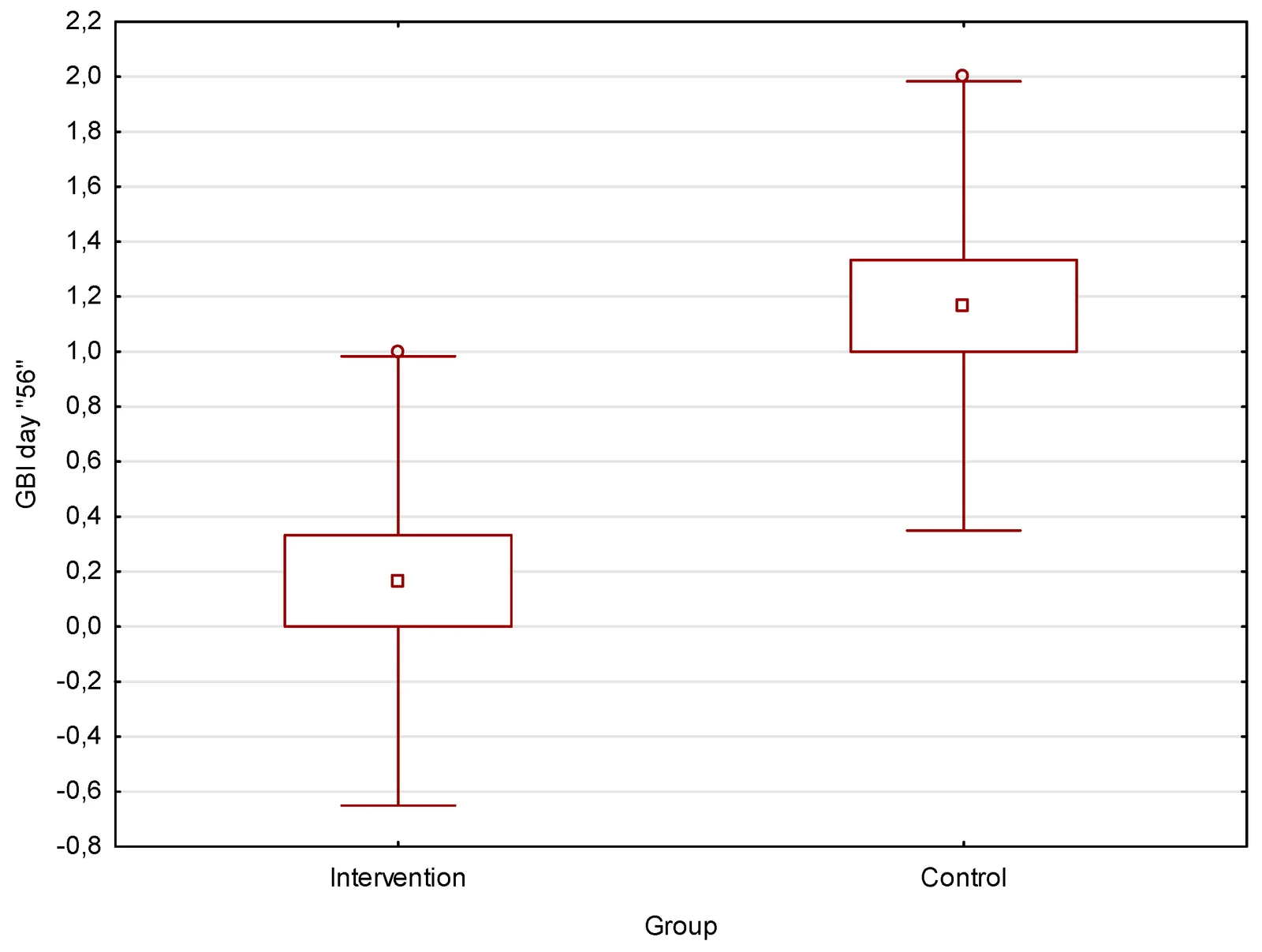
Boxplot showing the distribution of Gingival Bleeding Index (GBI) values in the control and probiotic-treated groups on Day 56 (D56), demonstrating the efficacy of probiotic supplementation.

**Figure 7:**
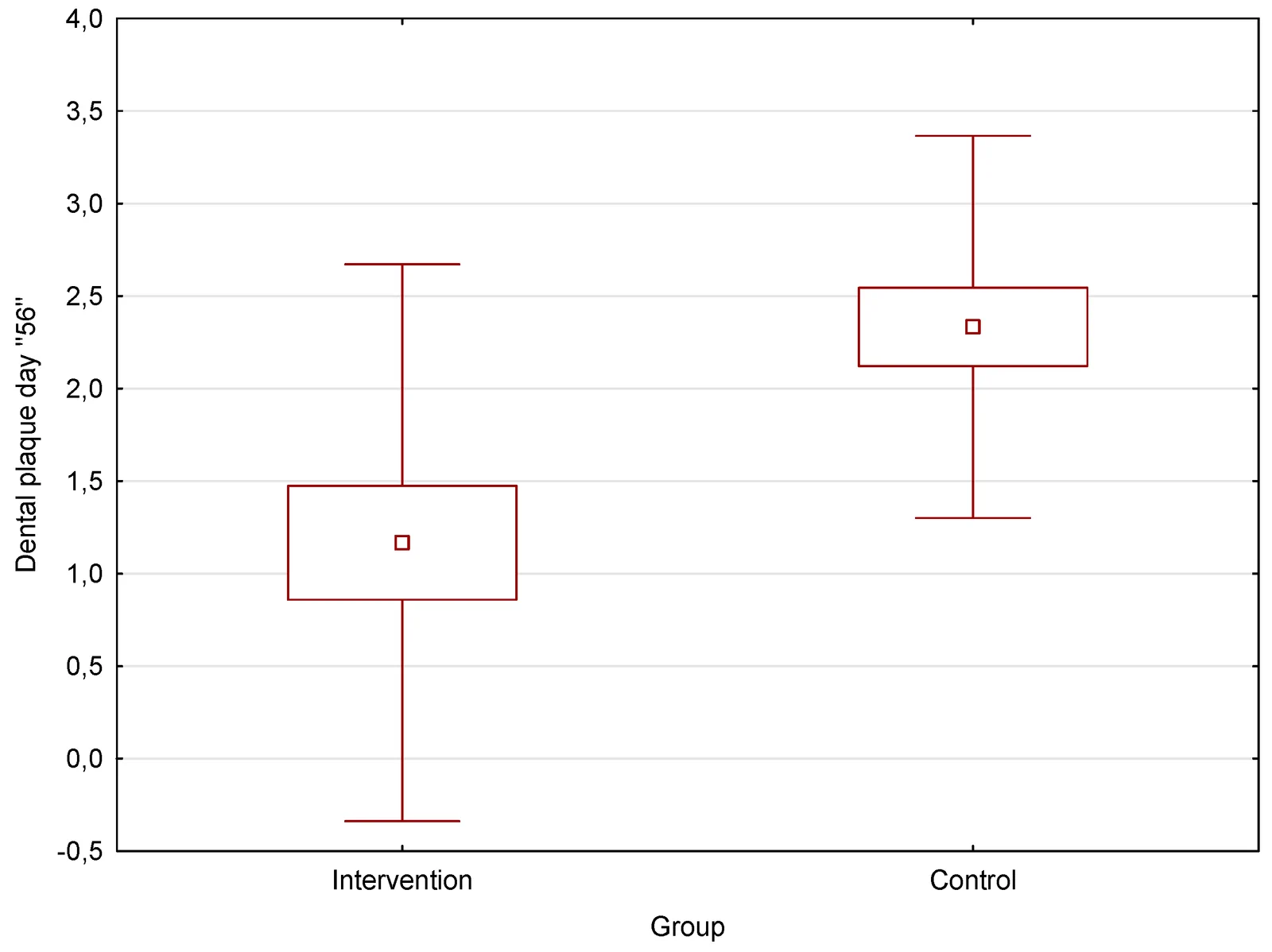
Boxplot showing the distribution of Dental Plaque Index values in the control and probiotic-treated groups on Day 56 (D56), demonstrating the efficacy of probiotic supplementation.

**Figure 8:**
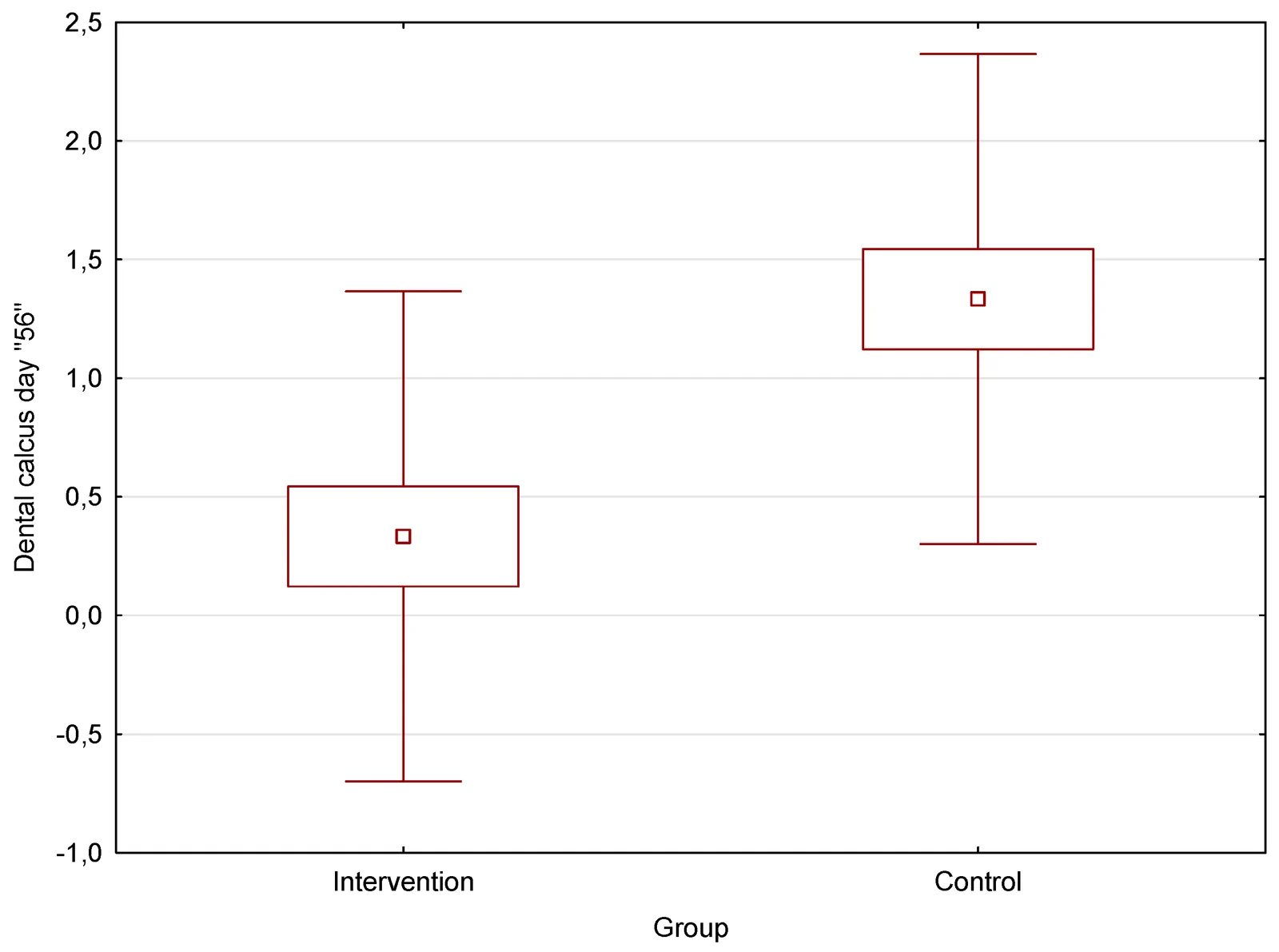
Boxplot showing the distribution of Dental Calculus Index values in the control and probiotic-treated groups on Day 56 (D56), demonstrating the efficacy of probiotic supplementation.

Statistically significant differences were observed between the groups for all evaluated oral health parameters. The Gingival Bleeding Index (GBI) was significantly lower in the probiotic-treated group than in the control group (*p* = 0.008) (Figure 6). Likewise, significant differences were observed for the Dental Plaque Index (*p* = 0.020) (Figure 7) and the Dental Calculus Index (*p* = 0.018) (Figure 8). These findings show that after 56 days of probiotic supplementation, the dogs receiving the probiotic preparation exhibited significantly better oral health than those in the control group.

## 4. Discussion

The canine oral cavity constitutes a highly complex ecosystem and represents the second-largest reservoir of microorganisms in the body, surpassed only by the intestinal microbiome [13,18]. Under physiological conditions, this microbial community remains in a state of homeostasis; however, numerous local and systemic factors may lead to dysbiosis, which is characterized by a loss of microbial diversity and an increase in the abundance of pathogenic microorganisms [19]. In dogs, this process is closely associated with the formation of dental plaque and dental calculus, which are the primary causes of periodontal disease [19]. At present, most strategies aimed at improving oral health in dogs focus exclusively on the mechanical removal of dental plaque. Research on biotic interventions in canine oral health remains at an early stage, with relatively few published interventional studies [19].

Maturation of the dental plaque biofilm is associated with the development of anaerobic and microaerophilic niches that promote the proliferation of periodontal pathogens [20,21]. One emerging direction in oral microbiome modulation is the use of postbiotics as agents to inhibit the growth of periodontal pathogens. Florit-Ruiz et al. [22] demonstrated that the postbiotic HT *Lactibacillus plantarum* CECT 9161 may be associated with reduced dental plaque accumulation and modulation of the oral microbiome. Their results indicated that the postbiotic supported oral health; however, the study did not evaluate the potential for microbiome transplantation using a postbiotic substrate or establish a causal relationship between the microbiome alterations and administration of *L. plantarum*. Here, we demonstrate the effectiveness of combining *L. plantarum* AMT4 and AMT14 with *Bifidobacterium animalis* AMT30 which significantly reduced the dental plaque and dental calculus on teeth. In dogs that did not receive probiotic supplementation following professional dental cleaning, although all parameters improved relative to baseline values, the results obtained on Day 56 indicate gradual re-accumulation of dental plaque and dental calculus, together with partial recurrence of gingival inflammation. While Florit-Ruiz et al. [22] documented sustained biofilm control using a single-strain postbiotic, our findings revealed a slight trend toward renewed plaque accumulation between Days 28 and 56 in the treated group (Table 2). This divergence can be attributed to differences in administration vehicles and the open-system design of our study. Unlike controlled laboratory settings, client-owned dogs exhibit significant behavioral and dietary variance, which can temporarily dilute the local concentration of orally administered probiotics during their transient mucosal contact, highlighting the clinical challenges of achieving permanent oral microbiome restructuring in domestic environments.

These results indicate that both groups were comparable with respect to all evaluated oral health parameters before initiation of probiotic supplementation. The absence of statistically significant baseline differences indicates that no distinct clinical disparities were detected between the groups prior to the intervention; however, given the small pilot sample size (*n* = 6 per group), this lack of significance does not mathematically establish absolute group equivalence or homogeneity.

Comparable clinical outcomes in canine passive oral hygiene were reported by Gawor et al. [23]., who evaluated a pomegranate extract drinking-water additive, noting a significant reduction in plaque and calculus scores over a 30-day period. However, while mechanical or plant-extract additives typically yield a 20% to 35% reduction in clinical indices, our biotic intervention demonstrated a more pronounced effect, with an 85.7% lower mean GBI and a 75.0% lower Dental Calculus Index compared to the control group on Day 56. This suggests that biotic interventions could potentially offer a distinct physiological approach compared to non-biological passive agents by interfering with early biofilm maturation rather than merely delaying mineral deposition, though direct microbiological verification is required to confirm this mechanism.

Although the mechanism of action of pomegranate extract is based primarily on its antibacterial and antioxidant properties, whereas probiotics exert their effects through biological competition and immunomodulation, both approaches may result in a similar clinical outcome, namely a reduction in oral dysbiosis. Therefore, the efficacy of probiotics appears to result not from direct bactericidal activity but rather from restoration of microbial homeostasis.

The mechanism of action of probiotics, such as the commercial preparation PetBIOM^®^ containing *Lactiplantibacillus* and *Bifidobacterium* strains used in the present study, targets several key processes. Competitive exclusion is the primary physical barrier, where *L. plantarum* effectively outcompetes early colonizers for mucosal and pellicle binding sites. Chemically, the production of bacteriocins and organic acids lowers the local microenvironment pH, directly disrupting the structural integrity of any established biofilm matrices. Furthermore, the inactivated strains provide heat-killed cellular structures and postbiotic metabolites that interfere with bacterial cell-to-cell communication (“quorum sensing”), thereby halting the expression of factors required for plaque maturation. Concurrently, these components induce local immunomodulation by downregulating pro-inflammatory cytokine cascades in the gingival epithelium, shifting the local ecosystem toward homeostasis [13,24].

With respect to dental calculus formation, previous studies have demonstrated substantial alterations in the oral microbiome during this process. In healthy dogs, Gram-negative bacteria, including Bergeyella and Porphyromonas, predominate [18,25]. As the biofilm matures and dental calculus undergoes mineralization, the relative abundance of Gram-positive bacteria, such as Bacillota and Actinomycetota, increases. These microorganisms produce larger quantities of extracellular polysaccharides, thus stabilizing the biofilm structure [19]. Probiotics may counteract these changes through coaggregation with pathogenic bacteria, which hinders their colonization of the tooth surface and may contribute to the reduction in clinical signs such as halitosis [24]. From a clinical perspective, the statistically significant *p*-values in this pilot study are directly translated into tangible health outcomes. An 85.7% lower mean Gingival Bleeding Index implies a profound reduction in localized vascular congestion and epithelial ulceration within the gingival sulcus. For practicing veterinarians, this degree of calculus and plaque restriction reduces the frequency of mandatory professional scaling under general anesthesia, thereby mitigating anesthetic risks for older canine patients and establishing a realistic non-invasive strategy for long-term home care.

An important aspect of probiotic activity is its influence on the epithelial barrier and the immune response. Strains of *Bifidobacterium animalis subsp. lactis* HN019 have been shown to strengthen epithelial tight junctions, thereby regulating epithelial permeability and preventing penetration of macromolecules capable of inducing inflammation [26]. Clinical studies have demonstrated that administration of this strain results in reduced gingival bleeding, decreased periodontal pocket depth, and lower concentrations of pro-inflammatory cytokines in humans [13].

Notably, probiotic interventions may reduce the abundance of major cariogenic pathogens, such as Streptococcus mutans, while modifying salivary pH, thereby protecting enamel against demineralization [27,28].

The oral cavity and the gastrointestinal tract remain functionally interconnected through the continuous swallowing of saliva, allowing for the constant passage of microorganisms from the oral cavity to the intestines [13]. Oral dysbiosis resulting from inadequate oral hygiene or advanced periodontal disease may disturb the composition of the intestinal microbiota and initiate a cascade of systemic inflammatory responses [12]. Likewise, the oral ecosystem may be affected by systemic disorders, such as gastroesophageal reflux disease, in which migration of gastric microbiota caused by reflux secondarily alters the microbial profile of the oral cavity [29]. Consequently, the beneficial effects of probiotic therapy on gastric and intestinal health may also indirectly contribute to improved oral health. However, the current evidence suggests that continuous and long-term probiotic supplementation is required to maintain these beneficial effects, as short-term microbiological changes tend to disappear after discontinuation of supplementation [1,2].

With the increasing prevalence of antimicrobial resistance in dentistry and veterinary medicine, probiotics represent an attractive alternative or adjunct to conventional therapeutic approaches [13,20,30]. Considering the complexity of the canine oral microbiota, including the presence of multidrug-resistant and potentially zoonotic bacteria, modulation of the oral microbiome through probiotic supplementation appears to be a promising strategy for the prevention and management of periodontal disease [25,31]. The results of the present study indicate that probiotics may represent a valuable adjunct to standard dental prophylaxis in dogs. Their use may be particularly beneficial in animals for which routine home oral hygiene is difficult or insufficient [6,28,31]. Several limitations must be acknowledged when interpreting these results. First, the sample size is relatively small (*n* = 12), which restricts the generalizability of the findings. Second, while all dogs were transitioned to an identical standardized diet during the 56-day trial to eliminate confounding dietary variables, their heterogenous nutritional histories prior to enrollment could have resulted in differences in baseline mucosal health parameters and initial plaque accumulation kinetics that were not measured. Third, clinical adherence relied partly on owner compliance logs and returned sachet counts, introducing a potential risk of reporting subjectivity. Fourth, a major methodological limitation of this trial is the complete absence of longitudinal microbiological data. Because no next-generation sequencing (NGS) or qPCR analyses were performed, this study did not directly evaluate changes in the overall oral microbial composition, the relative abundance of specific periodontal pathogens, or the downstream viability and persistence of the administered bacterial strains. Consequently, while the visual scoring indices demonstrated significant improvements, these clinical findings remain associative and cannot establish the definitive microbiological or molecular mechanism by which the probiotic preparation exerted its beneficial effects. Finally, the absence of investigator blinding during the clinical scoring introduces a potential risk of observer bias, which should be mitigated in future trials by employing independent, blinded evaluators [6].

## 5. Conclusions

In conclusion, this pilot study indicates that the daily oral administration of a probiotic preparation with transient oral exposure containing *Lactiplantibacillus* and *Bifidobacterium* strains following a professional dental procedure may aid in reducing dental plaque accumulation and alleviating mild gingival inflammation in a small cohort of dogs. These preliminary findings suggest that biotic modulation could serve as a supportive tool in canine dental prophylaxis, particularly when mechanical tooth brushing is not feasible. However, due to the pilot nature and limited sample size of this study, these results should be interpreted with caution. Larger, randomized, double-blinded clinical trials are required to definitively establish efficacy and form concrete clinical recommendations before this protocol can be widely adopted in veterinary guidelines.

## Figures and Tables

**Table 1:** Descriptive statistics of oral health parameters in the control group relative to baseline. The data are presented as the mean, median, minimum, maximum and standard deviation. *n* = 6.

Variable	Mean	Median	Minimum	Maximum	Standard Deviation
GBI (Day 0)	2.17	2.00	1.00	3.00	0.75
GBI (Day 28)	0.50	0.50	0.00	1.00	0.55
GBI (Day 56)	1.17	1.00	1.00	2.00	0.41
Dental Plaque (Day 0)	4.00	3.50	3.00	6.00	1.26
Dental Plaque (Day 28)	1.67	1.50	1.00	3.00	0.82
Dental Plaque (Day 56)	2.33	2.00	2.00	3.00	0.52
Dental Calculus (Day 0)	2.50	2.50	2.00	3.00	0.55
Dental Calculus (Day 28)	0.67	0.50	0.00	2.00	0.82
Dental Calculus (Day 56)	1.33	1.00	1.00	2.00	0.52

**Table 2:** Descriptive statistics. Data are presented as mean, median, minimum value, maximum value, and standard deviation. *n* = 6.

Variable	Mean	Median	Minimum	Maximum	Standard Deviation
GBI (Day 0)	1.83	2.00	1.00	3.00	0.75
GBI (Day 28)	0.17	0.00	0.00	1.00	0.41
GBI (Day 56)	0.17	0.00	0.00	1.00	0.41
Dental Plaque (Day 0)	4.00	4.00	3.00	6.00	1.10
Dental Plaque (Day 28)	0.50	0.50	0.00	1.00	0.55
Dental Plaque (Day 56)	1.17	1.00	0.00	2.00	0.75
Dental Calculus (Day 0)	2.50	2.50	2.00	3.00	0.55
Dental Calculus (Day 28)	0.33	0.00	0.00	1.00	0.52
Dental Calculus (Day 56)	0.33	0.00	0.00	1.00	0.52

**Table 3:** Statistical analysis of baseline comparability between the control and probiotic-treated groups (D0). *n* = 6.

Variable	Rank Sum (Control Group)	Rank Sum (Study Group)	U	Z	*p*
GBI (Day 0)	34.50	43.50	13.50	−0.64	0.487
Dental Plaque (Day 0)	40.00	38.00	17.00	0.080	0.932
Dental Calculus (Day 0)	39.00	39.00	18.00	−0.080	0.927

**Table 4:** Statistical comparison of the Gingival Bleeding Index (GBI), Dental Plaque Index, and Dental Calculus Index between the probiotic-treated and control groups on Day 56 (D56). *n* = 6.

Variable	Rank Sum (Control Group)	Rank Sum (Study Group)	U	Z	*p*
GBI (Day 56)	23.50	54.50	2.50	−2.40	0.008
Dental Plaque (Day 56)	25.00	53.00	4.00	−2.16	0.020
Dental Calculus (Day 56)	25.00	53.00	4.00	−2.16	0.018

## Data Availability

The data presented in this study are available on request from the corresponding author. The data are not publicly available due to privacy and ethical restrictions.
